# Why Do Patients Move from Online Health Platforms to Hospitals? The Perspectives of Fairness Theory and Brand Extension Theory

**DOI:** 10.3390/ijerph16193755

**Published:** 2019-10-06

**Authors:** Wei Le, Po-Ya Chang, Yu-Wei Chang, Jiahe Chen

**Affiliations:** 1 College of Economics and Management, China Jiliang University, Hangzhou 310018, Zhejiang; 2 Key Laboratory of Quality Infrastructure Efficacy Research, AQSIQ, Beijing 100028, China; 3 Department of Leisure Industry and Health Promotion, National Taipei University of Nursing and Health Sciences, Taipei City 112, Taiwan; 4 Department of Business Management, National Taichung University of Science and Technology, Taichung City 404, Taiwan; 5 Department of Computer Science, Hong Kong Baptist University, Hong Kong, China

**Keywords:** eHealth, online healthcare services, fairness theory, brand extension theory, O2O commerce

## Abstract

Online healthcare services are growing rapidly. Patients can receive health support through communication with online health professionals. However, previous research on eHealth has focused on patients’ online behavioral intentions. The purpose of this study is to investigate the effect of online patient–doctor communication on offline behavioral intentions and to propose a conceptual model for eHealth. We integrate fairness theory and band extension theory to develop a research model. This is further tested by structural equation modeling (SEM), with 279 valid sets of data from patients on the online health platform. Using partial least squares analysis (PLS), the results show that perceived fairness is an important factor affecting online satisfaction and the willingness to ask online doctors, which in turn has a positive impact on the willingness to go to offline doctors. These findings shed light on the research model for eHealth and offer suggestions for improving patients’ online satisfaction, as well as both online and offline behavioral intentions.

## 1. Introduction

With the development of the Internet, online healthcare services have become an emerging industry. Online healthcare services enable patients to access health support from online health professionals using information and communication technologies (ICT), such as blogs, online communities and forums, and patient–doctor interaction systems [1]. According to the report of Apple (2017), there were more than 5000 eHealth applications or pieces of software available globally to provide online health services. For example, Chunyu Yisheng (Spring Rain Doctor) is one of the most popular online health platforms in China, with more than 500,000 online doctors and 200 million users [2]. The market revenue of online healthcare services reached USD 179.6 billion in 2017 and is expected to reach USD 536 billion by 2025 [3].

Using online health platforms, a patient can easily interact with his/her doctor and get professional guidance. The interactive process determines patient online satisfaction and behavioral decisions about the online health platforms. Therefore, online communication with health professionals is a pertinent research topic. If a patient deems that online counseling is helpful and rational, he/she is likely to seek further treatment of the same health issues from an identical doctor in an offline hospital. On the other hand, if the patient is satisfied with the diagnosis or treatment of the offline doctor, he/she may be more willing to return to the online health platform to converse with identical doctors when he/she encounters a similar health problem. As such, the patient can save on search costs and does not need to spend extra time searching for a suitable doctor. Drawing on the advantages of breaking down geographical and temporal barriers, the complementation of these two patterns (i.e., from online to offline behavioral intentions and from offline to online behavioral intentions) can lead to a sustainable development of eHealth. The former is Online to Offline commerce, while the latter is Offline to Online commerce. According to the aforementioned discussion, this study aims to develop a conceptual model for sustainable eHealth.

Prior research on eHealth has only focused on online behavioral intentions [4,5,6]. In addition, a few studies have examined the effect of patient–patient communication on patients’ intention to visit offline doctors and emphasized the important roles of experience sharing and word-of-mouth between patients when patients choose offline hospitals and doctors [5,6]. Recently, with the development of the Internet and ICT, research on eHealth has been extended from patient–patient communication to patient–doctor communication. Nevertheless, no works have explored the impact of online patient–doctor communication on patients’ online and offline behavioral intentions. To the best of our knowledge, this study is the first to verify the research mode for sustainable eHealth based on online-to-offline commerce.

To fill this research gap, this study integrates fairness theory and brand extension theory to develop a research model. Given that the four sub-dimensions of fairness perception (i.e., distributive fairness, procedural fairness, interactional fairness, and informational fairness) can measure an individual’s comprehensive evaluation of online services [7], fairness theory explains the relationships between online patient–doctor communication and behavioral intentions. Additionally, the theory can be explained by social exchange theory, because patients pay money and spend time when asking for and receiving the expertise of online doctors. Considering that brand extension has been widely used to explain interrelations between multiple channels [8], brand extension theory is applied in this study to examine the relationship between online and offline behavioral intentions. Brand extension theory attempts to explain the positive impact of brands when a product enters a new market [8]. Consequently, if the patient is satisfied with the online doctor’s consulting services, he/she is more likely to converse with the same doctor again on the online health platform. On the other hand, in online-to-offline commerce, the patient is likely to be inclined to follow online doctors’ suggestions and visit an identical doctor for further treatment of the same health issue in an offline hospital, when he/she is satisfied with online counseling.

Drawing on these two theories, this study explores how online patient–doctor communication affects patients’ online satisfaction, as well as their online and offline behavioral intentions, and whether online satisfaction influences online and offline behavioral intentions. Based on the results in the introduction, online patient–doctor communication could be improved, which would in turn affect the online and offline relationships between doctors and patients. Doctors can increase patients’ online satisfaction and willingness to ask online doctors by affecting patients’ perception of fairness, which in turn increases patients’ willingness to go to offline doctors.

The remainder of this paper is organized as follows. Section 2 presents a literature review covering fairness theory and brand extension theory and develops the research model and hypotheses. Section 3 explains the data collection method and the measurements of each variable. Section 4 summarizes the results and the empirical findings are then discussed in Section 5. The theoretical and practical implications are provided in Section 6. The final section introduces the conclusions and limitations.

## 2. Literature Review and Hypotheses

### 2.1. A Conceptual Model for Sustainable eHealth

**Figure 1:**
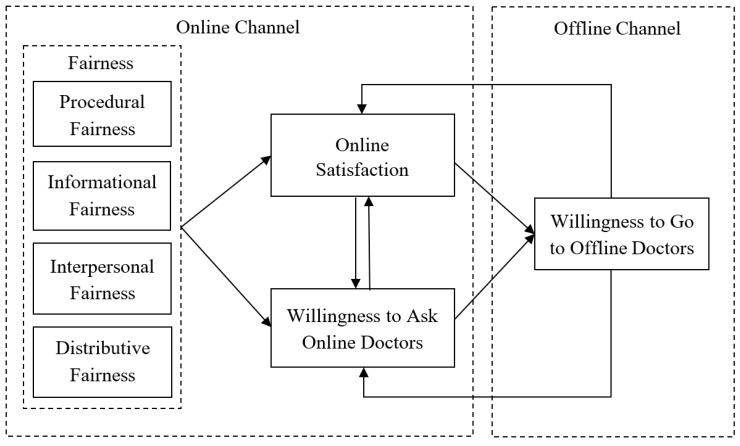
Conceptual model for sustainable eHealth.

Based on online-to-offline commerce, this study proposes a conceptual model for sustainable eHealth. As shown in Figure 1, fairness factors can measure patients’ evaluation of online consulting services based on four dimensions (i.e., procedural fairness, informational fairness, interpersonal fairness, and distributive fairness) [7]. Fairness factors have been confirmed to be important factors affecting an individual’s positive emotions and behavioral intentions [9,10]; in the same vein, if patients’ perceptions of online services are positive, fairness factors can promote patients’ satisfactory feelings and form online behavioral intentions. In the literature, satisfaction has been treated as a critical mediator of relationships with individual beliefs and behavioral intentions. In addition, a few researchers have revealed significant interactions between multiple channels [11,12], so this conceptual model proposes that online satisfaction and online behavioral intentions can interact with each other and further influence patients’ offline behavioral intentions. In turn, patients’ offline behavioral intentions may also have impacts on their online satisfaction and online behavioral intentions. Therefore, due to the interactions with online and offline channels, eHealth will have a dynamic and sustainable cycle which promotes the interactive relationship between these two channels.

Online health platforms such as Haodaifu (Good Doctor) and Chunyu Yisheng (Spring Rain Doctor) are increasingly being built to attract patients, doctors, and hospitals. These platforms integrate information and services provided by hospitals and doctors to help patients search for suitable doctors and consult online doctors. Similar to the effect of health webpages on offline behavioral intentions, the method of patients’ online communications with doctors might affect patients’ offline choices. To the best of our knowledge, no study has investigated the effect of online communications with doctors on offline behavioral intentions.

This study investigates patients’ communications with doctors on online health platforms, which allow patients to converse with online doctors. Patients’ assessment of the counseling might influence their choice to go to the doctors offline. We introduce fairness theory to explain patient assessment and integrate brand extension theory to illustrate the relationship between online patient–doctor communication and offline behavioral intentions. 

### 2.2. Fairness Theory

Fairness theory is related to equity in the sociological literature. An individual’s investment should be proportional to the rewards he/she receive [13]. Farkas and Anderson [14] have suggested developing a multidimensional approach to measure human exchange. Initially, distributive fairness [13,15,16,17] and procedural fairness [18,19,20] were proposed as the primary constructs. Later, researchers extracted another dimension from procedural fairness, namely, interactional fairness [21]. Greenberg [22] then proposed separating interactional fairness into interpersonal fairness and informational fairness. Therefore, four dimensions of fairness were finally extracted: distributive, procedural, interactional, and informational fairness. Colquitt [7] also posited the four-factor model of fairness, which has been identified to more closely match human behaviors.

In the e-commerce environment, fairness theory was used to affect the willingness to repeat purchases when Chiu et al. [23] investigated online auctions. Fu et al. [24] determined that perceived fairness had an effect on consumer satisfaction and the intention to engage in electronic word-of-mouth communication. Additionally, Wu [25] and Wu et al. [26] studied complaint intentions in online shopping and verified that fairness was a key element in promoting customer satisfaction and the intention to complain. In community health, fairness plays an important role in individuals who respond to decision-making processes and outcomes [27,28,29].

With the development of the Internet, medical counseling has been integrated into online-to-offline commerce. In online health services, patient–doctor communication can be regarded as an exchange behavior because patients spend time communicating with doctors and hope to get health knowledge from doctors. Because fairness theory can explain human exchange behavior [30], this study uses this theory to explain the behaviors of doctors and patients. If patients are satisfied with doctors’ online services, they are more likely to follow the advice of online doctors and visit identical doctors offline for further treatment or diagnosis of the same health issues. Therefore, this study posits that fairness theory can be adapted to investigate patients’ online and offline behavioral intentions. 

In an online environment, doctors might actively respond to requests and provide a diagnosis and prescription for patients based on their medical ethics [31]. Moreover, patients are likely to be concerned about whether they are respected and whether the doctors communicate candidly. These factors can be accounted for by the aforementioned four fairness dimensions. 

Distributive fairness is defined as an assessment of the fairness of economic and socioemotional outcomes. In general, a patient expects appropriate results and valuable information after asking an online doctor. If the patients are satisfied with the offerings provided by the doctor, and if the service meets their expectations, their perception of distributive fairness will increase, which in turn further promotes the overall fairness assessment [23].

Procedural fairness is defined as the perception of the fairness concerning the procedures of online counseling. Interpersonal fairness is defined as the extent to which a patient is treated with politeness, dignity, and respect. Informational fairness is defined as an explanation provided to clarify the reasoning behind the counseling. Thorough and reasonable questions are likely to enhance the overall perceived fairness. Previous scholars have validated that the overall perceived fairness is formed by distributive fairness, procedural fairness, interpersonal fairness, and informational fairness [23]. Therefore, we hypothesize the following:

**Hypothesis** **1a (H1a).** *Procedural fairness is positively related to perceived fairness;*

**Hypothesis** **1b (H1b).** *Informational fairness is positively related to perceived fairness;*

**Hypothesis** **1c (H1c).** *Interpersonal fairness is positively related to perceived fairness;*

**Hypothesis** **1d (H1d).** *Distributive fairness is positively related to perceived fairness.*

Satisfaction is defined as a patient’s positive emotions about the doctor. When a patient is served in an impartial manner during the counseling, he/she is more likely to consider consulting from the same online doctor again in the future. Fairness has been shown to positively influence satisfaction [24,25,26,32] and behavioral intentions [23,27,28,29,33]. An increase in online satisfaction will enhance the patient’s willingness to ask online doctors in the future. Studies have shown that satisfaction is positively associated with continued intentions [34,35,36,37,38]. Therefore, we hypothesize the following:

**Hypothesis** **2 (H2).** *Perceived fairness is positively related to online satisfaction;*

**Hypothesis** **3 (H3).** *Perceived fairness is positively related to the willingness to ask online doctors;*

**Hypothesis** **4 (H4).** *Satisfaction is positively related to the willingness to ask online doctors;*

### 2.3. Brand Extension Theory

Brand extension is a marketing strategy that provides a way to leverage an established brand identity. This strategy reduces the risk and increases the sales efficiency when a certain product enters a new market [8]. The associative network memory model is a recognized conceptualization of memory structures and has been applied in the brand literature. It posits that semantic memory or knowledge comprises a set of nodes and links. A node can activate other nodes through their association. This means that a brand, which is conceptualized as a node in memory, can be linked to various associations [39]. Therefore, when a brand is closely linked with a positive image, even if the product is strange to the customers, it becomes a potential source of activation for the image node.

Brand extension theory has been widely used in multichannel environments. Scholars have adopted this theory to examine the relationships between online and offline channels [8,40,41]. For instance, Yang et al. [42] reported that the offline channel service quality had a positive influence on the perceived online channel service quality on the basis of the channel extension mechanisms. Similarly, Kwon and Lennon’s [43] research on online loyalty revealed that offline images had a considerable impact on online images in the retail environment.

The effect of online behavioral intentions on offline behavioral intentions can be explained by an extension mechanism. In an online environment, doctors can build a brand image through online health platforms. In order to enhance the brand image, doctors are increasingly providing online health services to patients. Subsequently, doctors’ online brand images may attract many patients to their physical hospitals. Therefore, online brand imagery enhances patients’ offline behavioral intentions, as well as online behavioral intentions. 

Considering the above, the relationship between online and offline behavioral intentions seems to be mutual. A patient who is satisfied with an online doctor is more likely to go to an offline doctor. Additionally, the higher the willingness to ask the online doctor, the higher the willingness to go to the offline doctor. Therefore, we hypothesize the following:

**Hypothesis** **5 (H5).** *Online satisfaction is positively related to the willingness to go to offline doctors;*

**Hypothesis** **6 (H6).** *The willingness to ask online doctors is positively related to the willingness to go to offline doctors.*

**Figure 2:**
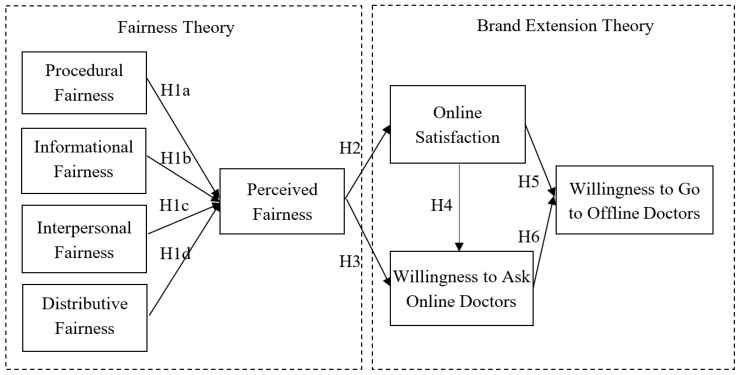
Research model for sustainable eHealth.

As shown in Figure 2, the research model of this study is presented below.

## 3. Methodology

### 3.1. Setting and Sample

This study assesses the process of online patient–doctor communication and evaluates how patients’ online and offline behavioral intentions are affected using patient data from the Chunyu Yisheng website, which provides online health services. The Chunyu Yisheng website was chosen because it is the earliest and largest online health platform in China. Patients can ask online doctors and further schedule an offline health service appointment with the doctors. In order to investigate respondents’ offline behavioral intentions, only respondents who asked doctors through the online health platform were included in the data analysis. With the assistance of doctors, 1200 online questionnaires were distributed to the patients using online health services.

### 3.2. Measures

To ensure the content validity, the items were developed based on previous research [7,44]. All items were measured by a seven-point Likert scale ranging from strongly disagree (1) to strongly agree (7).

Distributive fairness was employed to assess the level of fairness of economic and socio-emotional outcomes using four items; for example, “The outcome reflects the effort I put into asking the doctor” and “The outcome is appropriate for the online counseling process I have completed”. Procedural fairness was used to assess a patient’s perceived procedural fairness using four items; for example, “During the online counseling, the doctor answered my request in a timely manner” and “During the online counseling, the doctor was flexible to respond to my concerns”. Interpersonal fairness was applied to assess the degree to which a patient was treated with politeness, dignity, and respect. It was elaborated as six items; for example, “The doctor refrained from improper remarks or comments” and “The doctor treated me with empathy”. Informational fairness was employed to assess a patient’s perceived informational fairness using five items; for example, “The doctor explained my counseling thoroughly” and “The doctor seemed to tailor communications to individuals’ specific needs”.

Online satisfaction was used to assess a patient’s satisfaction with online doctors using five items; for example, “The online counseling makes me feel good” and “Overall, the online counseling makes me feel satisfied”. Willingness to ask doctors was used to assess a patient’s willingness to ask an online doctor using six items; for example, “I will consult the doctor on a regular basis in the future” and “I will frequently consult the doctor in the future”. Willingness to go to offline doctors was applied to assess a patient’s willingness to go to the doctor in hospitals using three items; for example, “I want to visit this doctor in the hospital in the future” and “It is likely that I will go to this doctor in the hospital in the future”.

### 3.3. Data Analysis

Partial least squares analysis (PLS) regression was used to evaluate the reliability and discriminant validity of the measurement model. To assess the reliability, the composite reliability (CR) and average variance extracted (AVE) were calculated [45]. The results of confirmatory factor analysis (CFA) for all items should be above the 0.70 loading criterion. In addition, two criteria were examined to assess the discriminant validity [46]. First, the loadings of the items on their respective constructs should be higher than the cross-loadings of the items on other constructs. Second, the square root of the AVE of each construct should be larger than its correlations with other constructs. In the structural model, PLS is used to test the research model and the results of hypothesis tests. Additionally, the significance of the paths is determined by using the T-statistic calculated with the bootstrapping technique. 

The measurement model and the structural model were tested using Smart PLS Graph version 3.00. PLS regression can analyze latent constructs modeled as formative and reflective. It was used in this study because the research model contains a formative construct, i.e., perceived fairness.

## 4. Results

### 4.1. Demographic Statistics

A total of 279 valid questionnaires were collected for data analysis. The response rate was 23.25%. The percentage of males and females was 54.8% and 45.2%, respectively. Most of the respondents had a university degree (66.7%). Respondents’ monthly income ranged from 1500 to 10,500 RMB (about US$ 200~1500) and most of respondents had incomes of 4501~6000 RMB per month (19.7%). All the respondents had experienced online counseling, and more than 45.5% of the respondents asked online doctors at least three times per month.

### 4.2. Measurement Model

This study used the convergent validity and discriminant validity to test the measurement model. The convergent validity was tested by the reliability, CR, and AVE [45]. The item reliability was confirmed by the factor loadings.

Table 1 details the results of the reliability. The reliability of each construct was assessed using Cronbach’s α. Cronbach’s α for each construct ranges from 0.84 to 0.94, exceeding the recommended value of 0.7 [47]. The CR for each construct ranges from 0.90 to 0.96, exceeding the recommended value of 0.7. The AVE for each construct ranges from 0.76 to 0.86, exceeding the recommended value of 0.5 [45]. Table 2 shows the results of the confirmatory factor analysis. The factor loading for each item ranges from 0.84 to 0.95, which exceeds the standard of 0.7 [48]. Table 3 shows that the correlations between the constructs range from 0.56 to 0.78, and the square root of the AVE of each construct is larger than its correlations with other constructs. Therefore, the convergence validity and discriminant validity are confirmed. 

### 4.3. Structural Model

**Figure 3:**
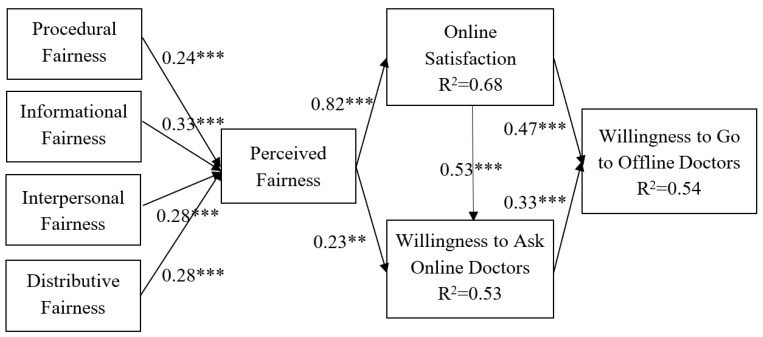
Results. ** *p* < 0.01; *** *p* < 0.001.

This study examined the SEM by testing the hypothesized relationships between nine variables (Figure 3). The results show that procedural fairness (β = 0.24, *p* < 0.001), informational fairness (β = 0.33, *p* < 0.001), interpersonal fairness (β = 0.28, *p* < 0.001), and distributive fairness (β = 0.28, *p* < 0.001) have a significant effect on the overall perceived fairness, supporting H1a to H1d. Perceived fairness (β = 0.28, *p* < 0.001) significantly affects online satisfaction. The model accounts for 68% of the variance in online satisfaction, supporting H2. Perceived fairness (β = 0.23, *p* < 0.01) and online satisfaction (β = 0.53, *p* < 0.001) have significant effects on the willingness to ask online doctors, supporting H3 and H4. The variables explain 53% of the variance in the willingness to ask online doctors. Satisfaction (β = 0.47, *p* < 0.001) and the willingness to ask online doctors (β = 0.33, *p* < 0.001) have a significant effect on the willingness to go to offline doctors, supporting H5 and H6. The variables explain 54% of the variance in the willingness to go to offline doctors.

## 5. Discussions

The results of this study show that the four dimensions procedural fairness, informational fairness, interpersonal fairness, and distributive fairness, positively affect the overall perceived fairness. This is consistent with previous studies [23]. In the context of eHealth, the findings imply that an increased efficiency, time and effort savings, accurate information, and interpersonal respect reflect patients’ overall perceived fairness. Likewise, as expected, the significant positive effect of perceived fairness on online satisfaction is validated, which confirms previous researchers’ suggestions [24,25,26,32]. In eHealth, the finding shows that if patients’ perceptions of online doctors’ consulting services are impartial, their satisfaction with online services will be strengthened. Perceived fairness and online satisfaction are found to have positive influences on the willingness to ask online doctors, which is also consistent with previous studies related to fairness theory [23,24,25,26,27,28,29,34,35,36,37,38]. These findings suggest that if patients are satisfied with online doctors’ consulting services, they will have strong intentions to converse with the same doctors again in the future. Additionally, if patients deem that they are treated fairly during online counseling, they will consult the same doctors again next time when they encounter similar health problems.

Based on the analysis results, we have also confirmed the hypothesized relationships that online satisfaction and the willingness to ask online doctors are powerful triggers for promoting the willingness to go to offline doctors. Although this confirms the suggestions of previous multichannel studies [8,40,41,42,43], the bulk of prior research on eHealth still heavily investigated the patients’ online behavioral intentions rather than the relationship between online and offline behavioral intentions. Based on brand extension theory, the result indicates that patients’ willingness to ask online doctors also directly affects their willingness to go to offline doctors. In the eHealth setting, the findings of this study elaborate that online doctors can establish their own brand images through delivering satisfactory online counseling to attract and maintain their patients online, or even promote patients’ visits to offline hospitals. Specifically, offline treatment in hospitals is a critical step for patients, so when patients are satisfied with online doctors’ consulting services, they are more willing to follow the doctors’ suggestions to visit the same doctors in an offline hospital for further treatment and diagnosis of the same health issues.

## 6. Implications 

### 6.1. Implications for Theory

Although online health services have been significantly studied, there is little exploration of the relationship between online patient–doctor communication and offline behavioral intentions. Currently, with the wide spread nature of the Internet and ICT, it is easier for patients to make contact with doctors through online health platforms. However, the fact that online health services have a greater impact on the patients’ willingness to go to offline doctors may have been ignored, because the bulk of academic attention has been paid to eHealth from only an online perspective [4,5,6]. This study investigates the effect of online patient–doctor communication on the willingness to go to offline doctors. Since online counseling is one mode of communication with health professionals, future research may investigate how other platforms, such as email and private social accounts, influence patients’ willingness to go to offline doctors.

A multi-theoretical model has been developed to discuss the whole process from online to offline behavioral intentions. Fairness theory and brand extension theory were integrated to explain two stages. The first stage is used to describe the interactive process using fairness theory. The second stage is employed to use the brand extension to explain the relationship between the willingness to ask online doctors and the willingness to go to offline doctors. Although the model was designed to show the patients’ behavioral intentions, it might be further applied to investigate multiple channels of consumer behaviors. 

Perceived fairness, which is formed by distributive fairness, procedural fairness, interpersonal fairness, and informational fairness, can assess interactive behavior. Many studies have verified the influence of fairness on behavioral intentions in commodity transactions or organization distribution [23,24,25,26,27,28,29]. This study has applied perceived fairness to examine online patient–doctor communication. Online counseling can be seen as an exchange behavior. A patient spends money to get professional knowledge from the doctor. According to the results, the perceived fairness is a key factor in determining the online satisfaction and has direct and indirect (through online satisfaction) effects on the willingness to ask online doctors. 

### 6.2. Implications for Practice

The study provides suggestions to help doctors communicate with patients and build their reputation to attract more patients.

First, brand effects can be extended from online to offline channels. If patients are willing to continue to ask an online doctor, they will have a stronger willingness to go to the doctor offline. As a result, this study suggests that doctors should strive to build a good image to improve patients’ online satisfaction and willingness to ask them. 

Second, patients’ online perception and experiences can significantly influence their online and offline behavioral intentions. Therefore, doctors should try to consider patients’ feelings and provide more thoughtful online counseling to meet the patients. For example, doctors should let patients know that online counseling is a good choice and beneficial to patients. 

Third, perceived fairness is the key factor in online satisfaction and the willingness to ask online doctors. According to the results of this study, the perceived fairness is related to all four dimensions, which are worth considering. Distributive fairness means a good outcome, which involves doctors’ professional ability. The outcome of online counseling should be as expected. Procedural fairness is about the experience during the process. Doctors should exactly respond to the questions asked during online counseling. Furthermore, during such communication, doctors should attach great importance to interpersonal fairness. Respect, empathy, and a detailed explanation will strongly influence patients’ feelings. In addition, doctors are encouraged to provide more helpful and valuable information to patients in need in a timely manner. Interactive fairness provides patients with a good experience, which in turn helps maintain a good brand image of doctors. In short, doctors should respect patients and interact with the patients politely.

## 7. Conclusions and Limitations

This study aimed to investigate factors affecting patients’ online and offline behavioral intentions, starting with online patient–doctor communication. According to fairness theory and brand extension theory, this study has proposed a conceptual model for sustainable eHealth. Based on the SEM, the results show that the greater the willingness to ask online doctors is, the greater the willingness to visit offline doctors is. Online satisfaction and the willingness to ask online doctors are influenced by the perceived fairness. As online health platforms have grown rapidly, this study is dedicated to enriching the sustainability literature and serves as the basis for sustainable commerce.

This study also has some limitations. First, the study used cross-sectional data to investigate and validate patients’ behavioral intentions instead of actual behaviors. Future studies are suggested to conduct a longitudinal survey to test patients’ actual behaviors. Second, the study samples were collected from the doctors, so patients who were dissatisfied may have refused to participate in the survey. Third, this study only emphasizes the role of online patient–doctor communication. Future research could focus on other influencing factors, such as social exchange factors and technological factors. Forth, the survey was conducted in China and the samples were collected from an online health platform. When generalizing the results to different countries or platforms, future research must pay attention to the differences between different cultures and platforms.

## Figures and Tables

**Table 1:** Results of the reliability test.

Construct	Mean	Std. Deviation	Cronbach’s α	CR	AVE
Procedural fairness	6.43	0.73	0.84	0.90	0.76
Informational fairness	6.43	0.75	0.89	0.93	0.76
Interpersonal fairness	6.40	0.80	0.89	0.93	0.82
Distributive fairness	5.94	1.05	0.88	0.93	0.81
Online satisfaction	6.38	0.83	0.94	0.96	0.85
Willingness to ask online doctors	6.01	1.07	0.94	0.96	0.82
Willingness to go to offline doctors	6.29	0.93	0.91	0.95	0.86

**Table 2:** Confirmatory factor analysis.

	PF	IFF	ITF	DF	SAT	WAD	WGD
PF1	**0.88**	0.66	0.56	0.58	0.62	0.54	0.50
PF2	**0.85**	0.66	0.56	0.60	0.55	0.47	0.51
PF3	**0.88**	0.71	0.59	0.57	0.58	0.46	0.47
IFF1	0.66	**0.83**	0.59	0.52	0.57	0.42	0.49
IFF2	0.70	**0.90**	0.66	0.64	0.61	0.49	0.56
IFF3	0.66	**0.86**	0.66	0.64	0.62	0.53	0.53
IFF4	0.68	**0.89**	0.75	0.71	0.67	0.53	0.52
ITF1	0.57	0.68	**0.87**	0.64	0.66	0.48	0.54
ITF2	0.62	0.70	**0.92**	0.72	0.71	0.58	0.55
ITF3	0.58	0.69	**0.93**	0.74	0.73	0.58	0.57
DF1	0.50	0.56	0.62	**0.84**	0.60	0.52	0.48
DF2	0.68	0.69	0.76	**0.95**	0.76	0.60	0.64
DF3	0.62	0.69	0.71	**0.92**	0.75	0.58	0.62
SAT1	0.61	0.64	0.71	0.72	**0.93**	0.66	0.69
SAT2	0.63	0.68	0.71	0.71	**0.93**	0.70	0.69
SAT3	0.64	0.66	0.72	0.72	**0.93**	0.65	0.60
SAT4	0.59	0.64	0.70	0.72	**0.89**	0.62	0.59
WAD1	0.54	0.52	0.56	0.60	0.67	**0.93**	0.61
WAD2	0.49	0.50	0.53	0.58	0.62	**0.92 **	0.60
WAD3	0.52	0.51	0.54	0.57	0.65	**0.93**	0.59
WAD2	0.49	0.51	0.56	0.55	0.64	**0.88**	0.55
WAD5	0.50	0.52	0.56	0.55	0.66	**0.85**	0.64
WGD1	0.52	0.54	0.56	0.58	0.62	0.61	**0.91**
WGD2	0.48	0.54	0.54	0.60	0.65	0.60	**0.93**
WGD3	0.56	0.60	0.59	0.62	0.68	0.63	**0.93**

Note: PF represents procedural fairness; IFF represents informational fairness; ITF represents interpersonal fairness; DF represents distributive fairness; SAT represents online satisfaction; WAD represents willingness to ask online doctors; WGD represents willingness to go to offline doctors. The bold indicators represent that factor loadings for each item of the corresponding construct are all higher than standard of 0.7 and larger than other factor loadings of other constructs.

**Table 3:** Inter-construct correlations.

	PF	IFF	ITF	DF	SAT	WAD	WGD
PF	**0.87**						
IFF	0.77	**0.87**					
ITF	0.65	0.77	**0.91**				
DF	0.67	0.72	0.77	**0.90**			
SAT	0.67	0.71	0.77	0.78	**0.92**		
WAD	0.56	0.57	0.61	0.63	0.72	**0.91**	
WGD	0.56	0.61	0.61	0.65	0.70	0.66	**0.93**

Note: The bold indicators represent that the square root of the AVE of each construct is larger than its correlations with other constructs.
